# Is additional balloon Kyphoplasty safe and effective for acute thoracolumbar burst fracture?

**DOI:** 10.1186/s12891-017-1753-4

**Published:** 2017-09-11

**Authors:** Ping-Jui Tsai, Ming-Kai Hsieh, Kuo-Feng Fan, Lih-Huei Chen, Chia-Wei Yu, Po-Liang Lai, Chi-Chien Niu, Tsung-Ting Tsai, Wen-Jer Chen

**Affiliations:** 1 Department of Orthopedic Surgery, Chang Gung Memorial Hospital and Chang Gung University, Taoyuan, Taiwan; 2 Bone and Joint Research Center, Chang Gung Memorial Hospital, Linkou, Taiwan; 3 5, Fu-Hsin Street, Kweishan Shiang, Taoyuan ,333, Linkou, Taiwan

**Keywords:** Burst fracture, Vertebroplasty, Kyphoplasty, Calcium sulfate/phosphate cement, Acute thoracolumbar burst fracture

## Abstract

**Background:**

Burst fracture is a common thoracolumbar injury that is treated using posterior pedicle instrumentation and fusion combined with transpedicular intracorporeal grafting after reduction. In this study, we compared the outcome of these two techniques by using radiologic imaging and functional outcome.

**Methods:**

Sixty-one patients with acute thoracolumbar burst fracture were operated with kyphoplasty (*n* = 31) or vertebroplasty (*n* = 30) and retrospectively reviewed in our institution between 2011 and 2014. All 61 patients underwent surgery within 5 days after admission to the hospital and then followed-up for 12 to 24 months after surgery.

**Results:**

Significant improvement was found in the anterior vertebral height (92 ± 8.9% in the kyphoplasty group, 85.6 ± 7.2% in the vertebroplasty group, *p* < 0.01) at 1 month post-operatively and (89 ± 7.9% in the kyphoplasty group, 78 ± 6.9% in the vertebroplasty group, *p* < 0.01) at the 24-month follow-up. Significant improvement was also observed in the kyphotic angle (1.2 ± 0.5° in the kyphoplasty group, 10.5 ± 1.2° in the vertebroplasty group, *p* < 0.01) at 1 month post-operatively and (5.4 ± 1.2° in the kyphoplasty group, 11.5 ± 8.5° in the vertebroplasty group, p < 0.01) at the 24-month follow-up. Both operations led to significant improvement of the patients’ pain and the Oswestry disability index (p < 0.01). Cement leakage was noted in 29% of patients after kyphoplasty and 77% of patients after vertebroplasty (p < 0.01). Only one implant failure (3.3%), which required further surgical intervention, was reported in the vertebroplasty group.

**Conclusions:**

Reduction with additional balloon at the fractured site is better than indirect reduction only by posterior instrumentation. The better reduction of kyphotic angle and the lower cement leakage rate in the kyphoplasty group indicate that additional balloon kyphoplasty is safe and effective for acute thoracolumbar burst fracture.

## Background

Burst fracture is a common thoracolumbar injury that occurs because of failure of the anterior and middle columns due to axial loading [1]. When anterior vertebral body height loss exceeds 50%, when the spinal canal is compromised by more than 50%, or when angulation is greater than 20° [1], surgical intervention should be considered. Recently, Thoracolumbar Injury Classification and Severity Score (TLICSS), which included fracture morphology, neurological injury and the integrity status of posterior ligamentous complex to determine stability and to decide operative or nonoperative treatment [2]. According to this classification, operative treatment is recommended for a score ≥ 5 points, and conservative nonoperative treatment for a score ≤ 3 points [2, 3].

Posterior pedicle instrumentation and fusion combined with transpedicular intracorporeal grafting after reduction is a well-known surgical technique [4, 5]. Several different types of grafts exist, including autogenous bone grafts, allogenous bone grafts, and artificial bone grafts; these bone grafts are composed of polymethylmethacrylate (PMMA) or calcium sulfate, and calcium phosphate, among others [6–8]. Because of the complications at the donor site with autogenous bone grafts [9] and the risk of infection with allogenous bone grafts, artificial bone grafts are now more widely used. In addition, studies have shown that artificial bone grafts are as efficient as autogenous bone grafts [10].

Balloon kyphoplasty is a relatively effective technique to reduce the fracture site [11–14]. Reduction by balloon is speculated to effectively correct vertebral body height and angle. Another advantage of balloon kyphoplasty is decreased cement leakage, which is often a problem with vertebroplasty. Few researches [15] have compared the effects of balloon kyphoplasty with traditional vertebroplasty in acute thoracolumbar burst fracture. Our study aimed to compare the radiologic and functional outcomes of these two techniques.

## Methods

Sixty-one patients with acute thoracolumbar burst fracture with or without neurologic deficit were treated with balloon kyphoplasty (*n* = 31) or vertebroplasty (*n* = 30) between 2011 and 2014 (Table 1). The Chang Gung Medical Foundation Institutional Review Board approved this study (103-3387B) and waived the requirement for informed consent because of the retrospective nature of the study. All the patients were treated with transpedicular intracorporeal grafting with calcium sulfate/phosphate cement and stabilization with pedicle-screw instrumentation. The results were compared in a retrospective, nonrandomized cohort study.

**Table 1:** Patient demographics data

Characteristic	Kyphoplasty	Vertebroplasty	*p* value
Number of patients	31	30	0.91
Age	35.5 ± 6.5	41.2 ± 6.8	0.814
Gender: female (%)	14 (54%)	12 (48%)	0.258
Location of fractured vertebrae			
Thoracic (T8–T10)	4	2	
T–L (T11–L1)aw	21	19	0.390
Lumbar (L2–L3)	6	9	
Neurological status (ASIA^**+**^)			
A	1	6	
B	0	2	
C	2	2	0.456
D	2	1	
E	26	19	
Hospital stay	12.5 ± 2.55	13 ± 3	0.551

ASIA^+^: American Spine Injury Association impairment scale [17]

Operation was indicated when the patient had a thoracolumbar injury classification and severity score [16] greater than 5. Additional balloon kyphoplasty was favored in the presence of kyphotic vertebrae and disruption of the posterior vertebral cortex, where higher-viscosity cement can be injected. All 61 patients underwent surgery within 5 days after admission to the hospital and were followed up for at least 24 months after surgery. Each patient underwent neurologic assessment by using a rating scale based on the American Spine Injury Association (ASIA) impairment scale [17]. The treatment outcomes were assessed with special reference to the anterior vertebral height (AVH), kyphotic angle, degree of back pain (visual analog scale or VAS), the Oswestry disability index (ODI) at pre-operative and post-operative follow-up after 1, 3, 6, 12, and 24 months. Complications including cement leakage and implant failure were also assessed. Beginning on the day after surgery and continuing for 3 months, patients were encouraged to ambulation while wearing a Taylor’s brace. Unrestricted activity was permitted after considering the individual patient’s neurologic situation.

## Operative technique

All operative procedures were performed with the patients under general anesthesia with endotracheal intubation. All patients were placed in a prone position on a radiolucent frame. Long or short constructs were planned and instrumented according to the level of the burst fracture. Long instrumentation (2 vertebrae above and 2 vertebrae below the fracture) was used for fractures between T11 and L2 (26 patients). Short instrumentation (One vertebra above and one vertebra below the fracture) was used if the fractured vertebra was located above T10 or below L3 (35 patients) (Table 2).

**Table 2:** Operative data

Operative Characteristic	Kyphoplasty	Vertebroplasty	*p* value
Short (1 above and 1 below fractured vertebra)	19	16	0.312
Long (2 above and 2 below fractured vertebra)	12	14	
Blood loss (ml)	265 ± 25	215 ± 85	0.058
Operative time (minutes)	125 ± 26	115 ± 14	0.25
Follow-up	29.5 ± 5.5	28.5 ± 4.5	0.462
Volume injected (ml)	12 ± 2.5	6.5 ± 1.5	<0.01

**Fig. 1:**
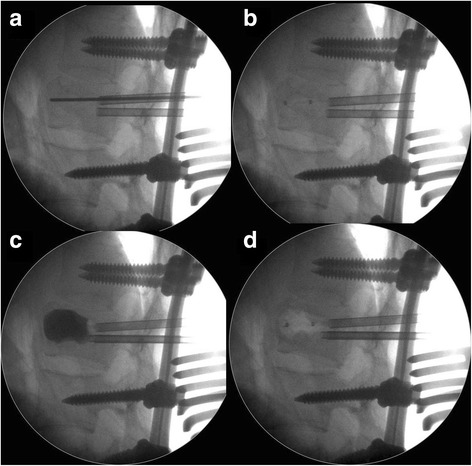
Radiographic image of kyphoplasty technique. Kyphoplasty was performed using a cannula and an expander, which were inserted into the pedicle, as well as a drill, which was inserted through the cannula. A needle pipe and pin was placed parallel to the superior endplate in the lateral view (**a**). The balloon was slowly inflated with initial bulk pressure (**b** and **c**). The volume of the balloon was carefully controlled to restore the fractured vertebra until adequate kyphotic angle reduction was obtained or the inflation pressure reached 220 psi [19]. The operator should record the amount of injected fluid to predict the cement volume. The balloon was deflated and withdrawn (**d**), and the created cavity was filled with cement [19]

**Fig. 2:**
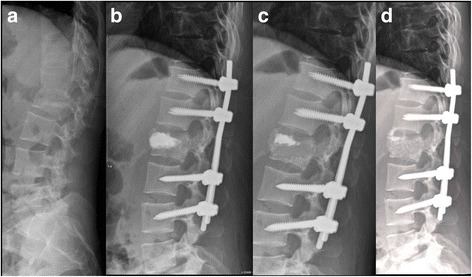
Radiographic image of one patient who received kypoplasty and long instrumentation. **a**. Lateral roentgenogram of a 25-year-old female patient showing a burst fracture of L2; the kyphotic angle is 30°. **b**. Postoperative lateral X-ray 3 days after surgery showing excellent restoration after kyphoplasty and long instrumentation; the kyphotic angle is −6°. **c**. Intact implant and kyphotic angle is −3° three months after surgery. **d**. Solid anterior fusion (continuous trabeculae of bridging anterior osteophytes without halo signs of screws) was achived and kyphotic angle was 0° 12 months after surgery

**Fig. 3:**
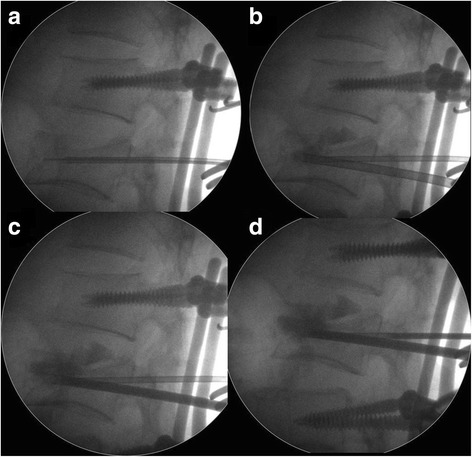
Vertebroplasty technique and cement leakage. Vertebroplasty was performed by inserting a cannula into the fractured vertebra (**a**). Calcium sulfate/phosphate cement was injected when the cannula tip reached the defect of the fractured vertebra under fluoroscopic monitoring (**b**, **c**). Intradiscal leakage and poor body reduction were noted during vertebroplasty (**d**)

**Fig. 4:**
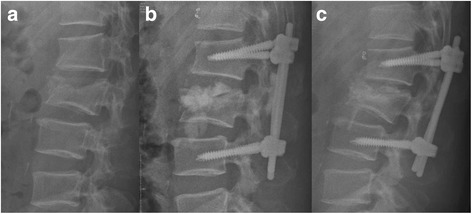
Radiographic image of one patient who received vetebralplasty and short instrumentation. **a**. Lateral roentgenogram of a 35-year-old male patient showing a L3 burst fracture; the kyphotic angle is 20°. **b**. Postoperative lateral roentgenogram showing good reduction (the kyphotic angle is 0 degree) through vertebroplasty and short instrumentation with intradiscal and paraspinal leakages 3 days after surgery. **c**. The kyphotic angle is −3° 12months after surgery and solid anterior fusion (continuous trabeculae of bridging anterior osteophytes without halo signs of screws) was achived. But the kyphotic angle is 12 degree and the rod was bended 12 months after surgery

Pedicle screws (SmartLoc® Spinal Fixation System, A-Spine Asia Co, Ltd., Taiwan) of the proper diameter were placed down into the pedicles of selected nonfractured vertebrae above and below the fractured vertebra using a standard posterior midline approach. Ligamentotaxis was achieved by proper longitudinal rod contouring [18] with distraction used along the entire area of instrumentation. Kyphoplasty was performed as follows: a cannula and an expander were inserted into the pedicle, and then a drill was inserted through the cannula. A balloon (Versys; SI Medical Co, Ltd., Korea) was inserted unilaterally into the fractured vertebral body and inflated slowly with initial bulk pressure [19]. Subsequently, the inflated balloon was deflated and withdrawn, then the intravertebral cavity was filled with calcium sulfate/phosphate cement (PRO-DENSE® Bone Graft Substitute, Wright Medical Technology, Arlington, TN, USA) [20]. This calcium phosphate cement product in a radiopaque liquid that was injected anteriorly into the void produced by the balloon inflation (Figs. 1, 2). Vertebroplasty was performed with a cannula inserted into the fractured vertebra. Under the fluoroscopic monitoring, the calcium sulfate/phosphate cement was injected when the cannula reached the defect of the fractured vertebra (Figs. 3, 4). Decompressive laminectomy was performed in patients with neurological deficit. Posterior or posterolateral fusions were not performed in either group. Union was defined as continuous trabeculae of bridging anterior osteophytes on plain films with absence of halo signs of screws. Probable fusion was defined as unclear trabecular bone with no radiolucent screws. Radiolucent screws or osteolysis of augmented artificial bone was labeled as nonunion [21]. Complications included cement leakages, wound infection, cardiopulmonary embolism and nonunion were recorded and analyzed. Cement leakages were classified into three types: type S, via the segmental vein; type B, via the basivertebral vein; and type C, through a cortical defect [22].

## Statistical analysis

Data from both groups were analyzed with SPSS statistical software package (SPSS Inc., Chicago, IL, USA). Tests of hypotheses between both groups were conducted using a Student t-test for numerical data (including age, follow-up period, operation time, estimated blood loss, AVH, kyphotic angle, presence of back pain [VAS], and the ODI). *P*-values <0.05 were defined as statistically significant.

## Results

No significant difference was observed in age, gender, location of fractured vertebrae, neurological status, and hospital stay in both groups (Table 1). According to the ASIA neurologic grading scale, 7 patients had grade A, 2 patients had grade B, 4 patients had grade C, 3 patients had grade D, and 45 patients had grade E. All 9 patients with incomplete neurologic deficit had at least 1 ASIA grade neurologic improvement at the 24-month follow-up.

The mean operative time in the kyphoplasty group was 125 min (range, 99–151 min) and 115 min in the vertebroplasty group (range, 101–129 min) which was not statistically significant difference(*p* = 0.25). The total volume of blood loss was 265 ml in the kyphoplasty group (range, 240–290 ml) and 215 ml in the vertebroplasty group (range, 130–300); the difference was also not statistically significant (*p* = 0.058). All patients were observed in an average follow-up of 29 months (range, 24–36 months). The volume of cement injected in the kyphoplasty group was 12 ml (range, 9.5–14.5 ml) and 6.5 ml in the vertebroplasty group (range, 5–8 ml); the difference was statistically significant (*p* < 0.01) (Table 2).

**Fig. 5:**
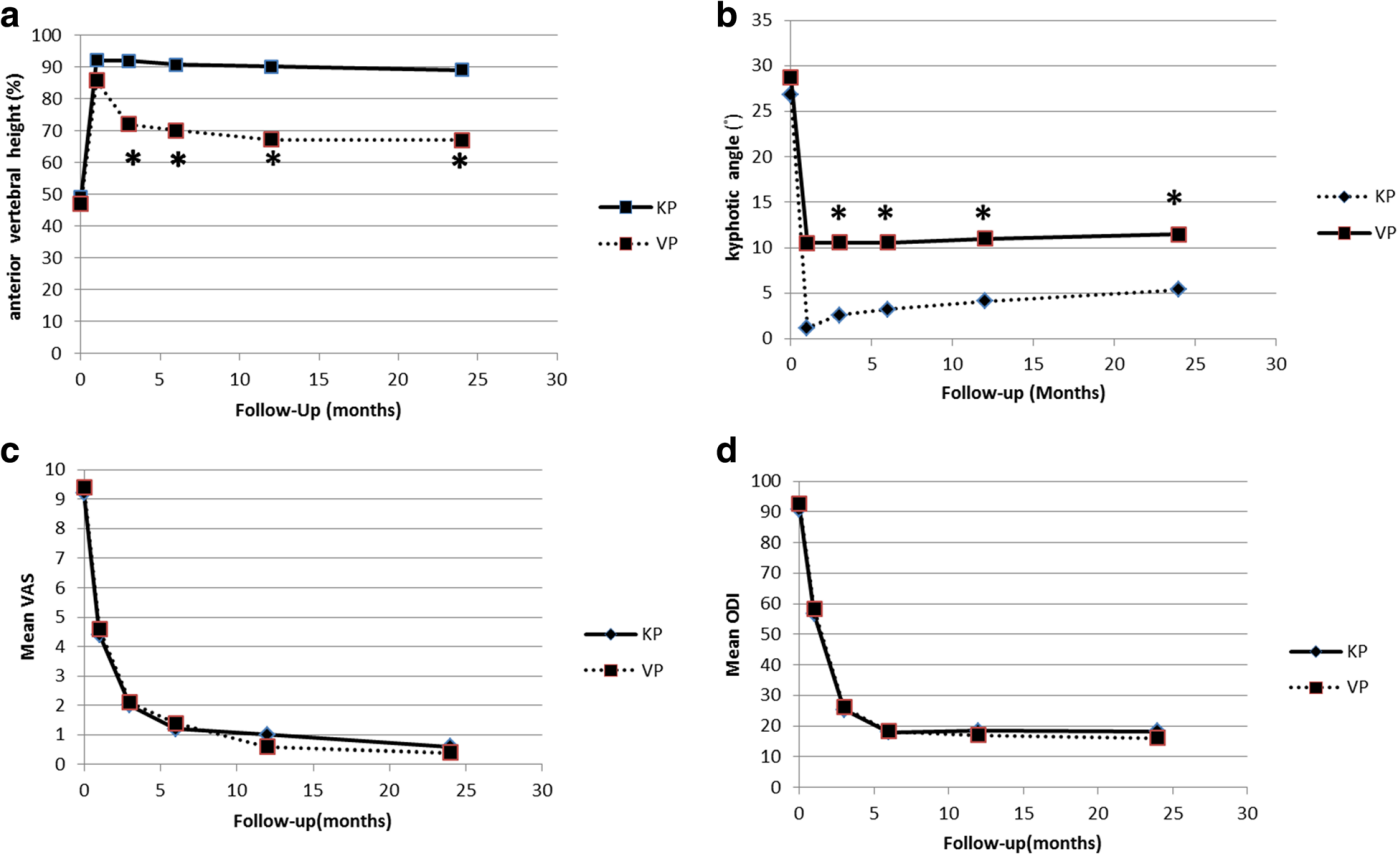
Comparisons of the kyphoplasty and vertebroplasty in anterior vertebral height (**a**), kyphotic angle (**b**), mean VAS (**c**) and ODI (**d**) at baseline and follow-up. Group calculated means and 95% confidence intervals are shown in panels A, B, C and D. In panels A and B, the treatment *p* value refers to the average treatment effect difference (*, *p* < 0.05) at 3, 6, 12, and 24 months follow-up. In panels C and D, no clinical treatment effect difference was found at pre-operative and post-operative follow-up

Significant improvement in the AVH (92 ± 8.9% in the kyphoplasty group, 85.6 ± 7.2% in the vertebroplasty group, p < 0.01) was found at 1 month post-operatively and (89 ± 7.9% in the kyphoplasty group, 78 ± 6.9% in the vertebroplasty group, p < 0.01) at the 24-month follow-up. The kyphotic angle between the two groups was also significant with 1.2 ± 0.5° in the kyphoplasty group and 10.5 ± 1.2° in the vertebroplasty group (*p* < 0.01) at 1 month post-operatively, and 5.4 ± 1.2° in the kyphoplasty group and 11.5 ± 8.5° in the vertebroplasty group (*p* < 0.01) at the 24-month follow-up (Fig. 5a, b). Both surgical procedures resulted in improvement of the patients’ pain and the ODI without statistical significance between the two groups (Fig. 5).

Complications.

**Fig. 6:**
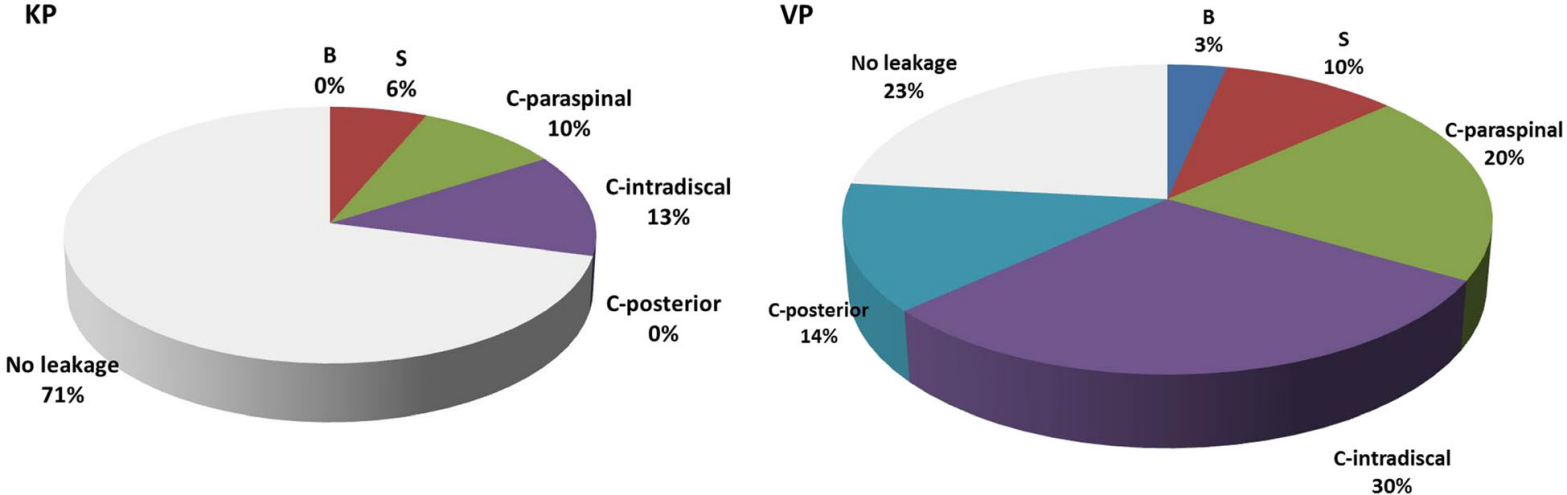
Comparisons of cement leakage between the kyphoplasty and vertebroplasty groups. Leakages of cement were classified into three different types [22]: type B, through the basivertebral vein; type S, through the segmental vein; and type C, through a cortical defect including three patterns in C-type leakage (i.e., paraspinal, intradiscal, and posterior). Kyphoplasty resulted in considerably less cement extravasation (29%) than vertebroplasty (77%). In our series, leakage into the spinal canal (C-posterior type leakage) is significant in the vertebroplasty group (14% in the vertebroplasty group to 0% in the kyphoplasty group)

The three patterns in C-type leakage are paraspinal, intradiscal, and posterior. Cement leakage was observed in 29% of patients in the kyphoplasty group (including 6% leakages via the segmental vessels, 10% through a paraspinal cortical defect, and 13% through a intradiscal cortical defect) and in 77% of patients after vertebroplasty (including 3% leakages via the basivertebral vein, 10% via segmental vein, 20% leakages through a paraspinal cortical defect, 30% through a intradiscal cortical defect, and 14% through the posterior cortical wall) (*p* < 0.01) (Fig. 6). There is no intraoperative cardiogenic hypotension, pulmonary embolism, or deteriorated neurological deficit in the 32 patients (9 and 23 in the kyphoplasty and vertebroplasty groups, respectively) with cement leakage. Superficial wound infection or deep wound infection was not found in our series. At the final follow-up, 60 of the 61 patients exhibited union or probable union. Nonunion was only in one implant (3.3%) in the vertebroplasty group, which required further surgical intervention. There is no adjacent vertebral fractures in the 2-year follow-up.

## Discussion

In this study, long instrumentation were performed in T11-L1 fractured vertebrae. 9 patients with T11-L1 fractured vertebrae in kyphoplasty group and 5 patients in vertebroplasty group were performed short instrumentation because of strong bone screw purchase. However, the results of many studies [10, 23–25] showed that lack of anterior support may lead to a high percentage of early implant failure. A large cavity in fractured vertebrae is created during posterior instrumented construct after application of a distraction force, and thus may lead to reduction site collapse and worsened clinical outcomes. The materials used for anterior reconstruction includes autogenous and artificial bone grafts. The results of several studies have shown that artificial bone grafts produce similar success rates to autogenous bone grafts. In addition, the use of artificial bone grafts may lead to shorter operation time and less blood loss [10].

Many techniques have been used to establish anterior vertebral support. Vertebroplasty, which was first used in 1987 [26], became an efficient method for treating compression fractures [27]. Balloon-assisted kyphoplasty is a developed technique [19, 28, 29] to restore the vertebral body height and to treat local kyphosis which also contributes to anterior column stability. In the present study, posterior screw fixation combined with kyphoplasty for anterior support produced significantly better kyphosis angle correction and better vertebral body height, as shown during the 24-month follow-up. Using posterior distraction, reduction of fractured vertebrae is improved indirectly by ligamentotaxis but distraction force also increases kyphotic angle. The distraction force applied in the kyphoplasty patients may be lower in the vertebroplasty patients, and thus post-operative lordotic angle may be better in the kyphoplasty group. The reported kyphotic angle correction with transpedicular bone grafting and short pedicle fixation techniques ranges from 64% to 105% [18, 30–33]. The average corrections of 92% in the kyphoplasty group and 85.6% in vertebroplasty group are within the previous reported range. The average loss of correction after posterior short segment instrumentation plus transpedicular augmentation with biodegradable bone cement is not significantly different from that following traditional anterior and posterior spinal operations [18, 30, 34], with the loss of correction ranging from 1° to 4.2°.

Posterior instrumentation is speculated to fail due to the large bone defect created inside the fractured vertebra after indirect height restoration through distraction and ligamentotaxis force applied during surgery [35]. Because the majority of patients with burst fractures in our series were aged between 29 and 48 years, the use of a more biocompatible bone cements (e.g.,calcium phosphate and hydroxyapatite) is preferred [30, 36, 37]. Self-hardening calcium sulfate/phosphate bone cements have been developed as alternatives to avoid long-term side effects of PMMA [38–40] because of the biocompatibility, no local heat or toxic effect on surrounding bone tissues [41, 42] and can stimulate bone formation at the bone–cement interface [43]. Korovessis P. [44] reported an improvement of kyphotic angle from preoperative 16° to postoperative 1° at final follow-up and the AVH ratio from 0.6 to 0.9 in 29 thoracolumbar burst fracture patients using kyphoplasty with calcium phosphate cement and pedicle screws instrumentation and reported a similar result in 18 thoracolumbar burst fracture patients [45] using balloon kyphoplasty with calcium phosphate cement combined with posterior short segmental fixation. In both of their study, they didn’t use vertebroplasty as a control group which is difficult to make a conclusion that the reduction using balloon at the site of the fractured vertebra is better than the indirect reduction by means of distraction provided only by instrumentation. We also doubt this similar data [44, 45] because of the distraction force applied in open surgery should be larger than minimal invasive method so that radiological result in open surgery should be better.

The reason why significant improvements were noted in kyphotic angle and AVH at post-operative one month, but decreased in the 24-month follow-up in our study, because of the calcium sulfate/phosphate cement was partial resorption.

In this study, the volume injected in the kyphoplasty group was significantly higher than that in the vertebroplasty group (12 ± 2.5 cm^3^ vs. 6.5 ± 1.5 cm^3^, *p* < 0.01) because the void space in fractured vertebrae was created by balloon kyphoplasty, and thus, the vertebral height and kyphotic angle will be better and maintained in the 24-month follow-up in the kyphoplasty group.

**Fig. 7:**
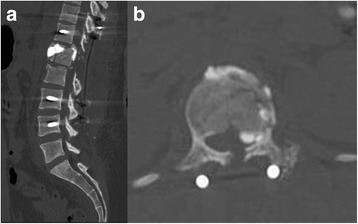
Cement leakage into the spinal canal after vertebroplasty. Vertebroplasty without balloon inflation causes cement leakage through the cortical disruption as detected on postoperative computed tomography scanning (**a**, **b**)

According to the previous studies, balloon assisted kyphoplasty results in considerably less cement extravasation, compared to vertebroplasty [46–48]. In a systematic review of clinical studies, the rate of cement leakage was 9% in kyphoplasty groups and 41% in vertebroplasty groups [49]. In a meta-analysis study, cement extravasation were observed in 7% of patients after kyphoplasty, but in 20% of patients after vertebroplasty [50]. In the present study, 29% of the kyphoplasty group had cement leakage, compared to 77% in the vertebroplasty group (Fig. 6). The higher leakage rate may be due to acute burst fracture in young patients instead of osteoporotic compression in older patients. The lower rate of cement leakage in the kyphoplasty group proved the fact that injection of high-viscosity cement at low pressure into a created cavity is a significant improvement over the injection of low-viscosity cement at high pressure into an unreduced fractured vertebra (Fig. 7) [19, 29, 49]. In our series, leakage into the spinal canal was significant in the vertebroplasty group (16% in the vertebroplasty group to 0% in the kyphoplasty group), indicating that kyphoplasty is a considerably safe procedure in the treatment of thoracolumbar burst fractures. Only one implant failure (3.3%), which required further surgical intervention, was found in the vertebroplasty group.

In our series, a larger amount of cement was injected into the vertebrae of the kyphoplasty group; this group exhibited better eventual stability than the vertebroplasty group. The authors believe that the reduction with additional balloon at the fractured site is better than the indirect reduction through distraction force provided by posterior instrumentation. Additional balloon kyphoplasty is safe and effective for acute thoracolumbar burst fracture.

There are several limitations in this study. 1) This is a retrospective study and mean follow-up was 29 months. The long-term effectiveness of this technique still needs to be evaluated. 2) The surgical indication is different in kyphoplasty group and vertebroplasty group. 3) The numbers of the patients in both groups were limited.

## Conclusions

Balloon kyphoplasty and vertebroplasty with injection of calcium sulfate/phosphate cement combined with posterior fixation for acute thoracolumbar burst fractures both provided immediate stability and reduction of post-traumatic segmental kyphosis. In addition, the use of additional balloon kyphoplasty led to better reduction of fractured vertebrae, less cement leakage, and better stability than in patients who only received vertebroplasty.
